# Toll-like receptor-2 deficiency induces schizophrenia-like behaviors in mice

**DOI:** 10.1038/srep08502

**Published:** 2015-02-17

**Authors:** Se Jin Park, Jee Youn Lee, Sang Jeong Kim, Se-Young Choi, Tae Young Yune, Jong Hoon Ryu

**Affiliations:** 1 Department of Life and Nanopharmaceutical Science, Kyung Hee University, Seoul 130-701, Korea; 2 Department of Oriental Pharmaceutical Science, College of Pharmacy, Kyung Hee University, Seoul 130-701, Korea; 3 Age-Related and Brain Diseases Research Center, School of Medicine, Kyung Hee University, Seoul 130-701, Korea; 4 Department of Biochemistry and Molecular Biology, School of Medicine, Kyung Hee University, Seoul 130-701, Korea; 5 Department of Physiology, Biomedical Science, College of Medicine, Seoul National University, Seoul 110-799, Korea; 6 Department of Physiology and Dental Research Institute, Seoul National University School of Dentistry, Seoul 110-749, Korea

## Abstract

Dysregulation of the immune system contributes to the pathogenesis of neuropsychiatric disorders including schizophrenia. Here, we demonstrated that toll-like receptor (TLR)-2, a family of pattern-recognition receptors, is involved in the pathogenesis of schizophrenia-like symptoms. Psychotic symptoms such as hyperlocomotion, anxiolytic-like behaviors, prepulse inhibition deficits, social withdrawal, and cognitive impairments were observed in TLR-2 knock-out (KO) mice. Ventricle enlargement, a hallmark of schizophrenia, was also observed in TLR-2 KO mouse brains. Levels of p-Akt and p-GSK-3α/β were markedly higher in the brain of TLR-2 KO than wild-type (WT) mice. Antipsychotic drugs such as haloperidol or clozapine reversed behavioral and biochemical alterations in TLR-2 KO mice. Furthermore, p-Akt and p-GSK-3α/β were decreased by treatment with a TLR-2 ligand, lipoteichoic acid, in WT mice. Thus, our data suggest that the dysregulation of the innate immune system by a *TLR-2* deficiency may contribute to the development and/or pathophysiology of schizophrenia-like behaviors via Akt-GSK-3α/β signaling.

Schizophrenia is a debilitating psychiatric disorder with a prevalence of 1% worldwide, and is characterized by positive symptoms, negative symptoms, and cognitive impairments^1^. Recent studies have revealed that neuronal abnormalities, including altered glutamatergic function, GABAergic deficits, altered dopaminergic function, and impaired neurodevelopment, contribute to the etiopathophysiology of schizophrenia^2^. Specially, growing evidences suggest that abnormal neurodevelopment during perinatal and early adolescence may lead to a dysfunction of neural networks which may later develop into a full-blown schizophrenic disorder^3^,^4^. Furthermore, dysregulation of the immune system, along with genetic and environmental factors, is known to contribute to abnormal neurodevelopment processes, which leads to the pathogenesis of schizophrenia^5^. For example, an imbalance in inflammatory cytokines, such as interleukins, interferon, tumor necrosis factor-α, and chemokines, has been implicated in the negative and cognitive symptoms of schizophrenia^6^,^7^. Prenatal immune activation is also known to induce schizophrenia-like behaviors in offspring, which are associated with abnormal neuronal development and synaptic transmission in an animal model of schizophrenia^8^. Thus, a dysregulation of the immune system appears to play an important role in the pathogenesis of schizophrenia.

A recent study has shown the close relationship between mental illness and the immune system, especially the innate immune system, in which a dysregulated immune system causes psychiatric disorders^9^. In general, the activation of TLR by pathogen-associated molecular patterns initiates an intracellular kinase cascade by inducing the translocation of transcription factor, NF-κB, which leads to the production of a variety of inflammatory mediators and cytokines^10^. Twelve functional TLRs have been identified in humans and mice, respectively, and they recognize different microbial ligands during infection^11^. In addition to the involvement in inflammatory processes, TLRs are also known to play important roles in neurodevelopment, adult neurogenesis, neuroplasticity, and learning and memory in the absence of any underlying immune activation^12^,^13^,^14^. For example, TLR-3 inhibits hippocampus-dependent working memory retention and adult hippocampal neurogenesis^12^. TLR-2 deficiency impairs hippocampal neurogenesis, whereas TLR-4 deficiency enhances neuronal cell proliferation and differentiation^14^. TLRs have also been shown to be involved in higher brain functions, such as cognition or mood-related behaviors^12^,^15^. However, there is no study demonstrating that the genetic mutation of TLRs may induce psychiatric disorder-like symptoms.

Here, we tried to examine whether the dysfunction of *TLR* genes such as *TLR-2*, *TLR-3*, or *TLR-4* by using knock-out (KO) mice would affect aggressive behavior, one of psychotic behaviors^16^. Surprisingly, we found that only TLR-2 KO mice, but not TLR-3 or TLR-4 KO mice, showed abnormal and aggressive behaviors in a preliminary experiment. Thus, to examine the abnormal behavior observed in TLR-2 KO mice in detail, we measured behavioral outcomes, histological alterations, and the level of neuroplasticity in TLR-2 KO mice. Furthermore, numerous studies have demonstrated the abnormalities in Akt (protein kinase B)-glycogen synthase kinase (GSK)-3 signaling in schizophrenia^17^,^18^. Therefore, we also investigated abnormalities of Akt-GSK-3 signaling and the effects of typical or atypical antipsychotic drugs such as haloperidol and clozapine on the abnormal signaling cascade and behavior in TLR-2 KO mice. Finally, we tried to elucidate the linkage between TLR-2 activation and Akt-GSK-3 signaling using lipoteichoic acid (LTA) as a TLR-2 ligand. Conclusively, we showed in this study that the deletion of the *TLR-2* gene in mice causes the typical behavioral, histological, and pathophysiological characteristics observed in schizophrenia patients.

## Results

### TLR-2 KO mice show hyperlocomotion and anxiolytic behavior

In our preliminary studies, we found that only TLR-2 KO mice exhibited abnormal and aggressive behavior; whereas these effects were not observed in TLR-3 and 4 KO mice (Supplementary Fig. 1a, b). Notably, the aggressive behavior of TLR-2 KO mice began to be observed apparently in 8 weeks old, whereas not in 3 or 5 weeks old (Supplementary Fig. 1c, d). Therefore, TLR-2 KO mice in 8–20 weeks old were employed in this study to examine behavioral, morphological, and biochemical characteristics.

**Figure 1:**
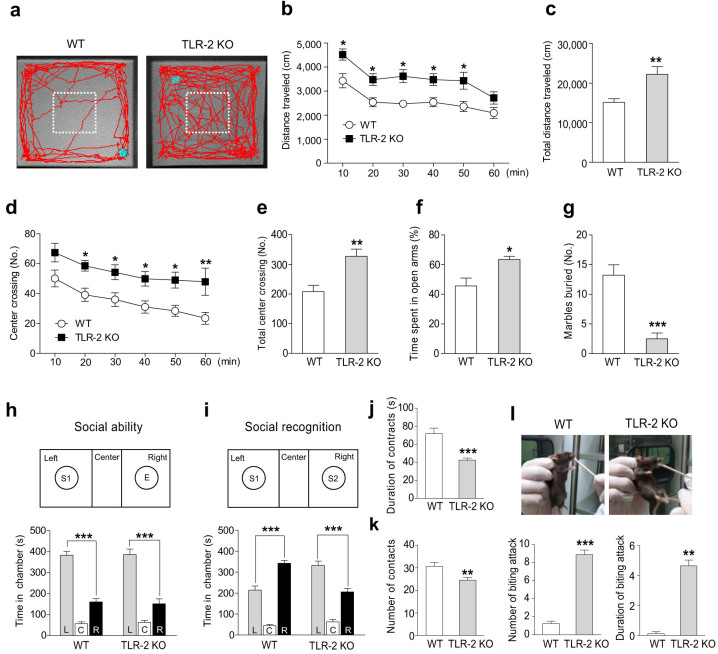
TLR-2 knock-out (KO) mice show hyperlocomotion, less anxiety-like behavior, social withdrawal, and aggressive behaviors. (a–e) Horizontal locomotor activity was measured using the open field test during 60 min. (a) Representative tracks for the open field test. (b–e) Distance traveled with 10 min interval (b), total distance traveled (c), number of center crossing interval with 10 min (d), or total number of center crossings (e) were significantly higher in TLR-2 KO mice (closed squares, *n* = 9) than wild-type (WT) mice (open circles, *n* = 10). The level of anxiety was determined using the elevated plus-maze (f) and marble-burying tests (g). TLR-2 KO mice spent more time in the open arms of the elevated plus-maze test (f; WT, *n* = 6; TLR-2 KO, *n* = 7) and showed less burying in the marble-burying test (g; WT, *n* = 16; TLR-2 KO, *n* = 12) compared with WT mice. (h, i) Three-chamber social novelty preference test. (h) When the stranger mouse was put in the left cage, both WT (*n* = 7) and TLR-2 KO (*n* = 8) mice spent more time in the left chamber. (i) When a second stranger mouse was placed in the right cage, WT mouse showed a strong preference for the new mouse, whereas TLR-2 KO mice did not. L, left; C, center; R, right; E, empty cage; S1, stranger mouse 1; S2, stranger mouse 2. In the reciprocal social interaction test, TLR-2 KO mice (*n* = 10) showed a decreased duration (j) and number of contacts (k) with the test mice compared to WT mice (*n* = 14). (l) In the cotton swab biting test, TLR-2 KO mice (*n* = 7) showed a significant increase in the number of biting attacks and the duration of attacks against the cotton swab compared to WT mice (*n* = 8). *P < 0.05, **P < 0.01, ***P < 0.001 vs. WT mice. All data are shown the mean ± s.e.m.

Although the three major symptoms of schizophrenia are difficult to replicate in rodents, hyperactivity in a novel environment has been used as a translational feature of psychotic agitation^19^. Thus, we first determined the spontaneous locomotor activity of TLR-2 KO and WT mice in an open field test. TLR-2 KO mice showed higher locomotor activity than WT mice (Fig. 1a). The distance traveled was also significantly longer in TLR-2 KO mice compared to WT mice (Fig. 1b, c). TLR-2 KO mice showed a marked increase in the number of crossings in the anxiety-provoking center of the open field compared to WT mice (Fig. 1d, e). Next, we conducted an elevated plus-maze test and a marble-burying test to confirm the anxiety-like behavior in TLR-2 KO mice. In the elevated plus-maze test, TLR-2 KO mice spent significantly longer times in the open arms of the maze (Fig. 1f) and entered open arms more frequently (Supplementary Fig. 2a) than WT mice, but no significant differences in locomotor activity were found (Supplementary Fig. 2b). In the marble-burying test, TLR-2 KO mice buried significantly less marbles compared with WT mice (Fig. 1g). However, TLR-2 KO mice did not show any change in immobility in the forced swimming test (Supplementary Fig. 3), which is used to detect anti-depressant-like activity. Collectively, these results indicate that TLR-2 KO mice show hyperlocomotion and reduced anxiety-like behaviors in a novel environment.

### Social function in TLR-2 KO mice

Next, we investigated whether TLR-2 KO mice would exhibit deficits in social function such as reduced social interaction as well as increased aggressive behavior, which are considered to negative symptoms of schizophrenia^20^. In the three-chamber social novelty preference test, both WT and TLR-2 KO mice had no preference for the two empty cages located in the right and left chambers during a habituation period (Supplementary Fig. 4). When the stranger mouse was put in the left cage, both WT and TLR-2 KO mice significantly spent more time in the left chamber (Fig. 1h), suggesting that there is no significant difference in the social ability between WT and TLR-2 KO. When a second stranger mouse was placed in the right cage, however, WT mice showed a strong preference for the new mouse, whereas TLR-2 KO mice did not (Fig. 1i), suggesting that social recognition may be impaired in TLR-2 KO mice. We next examined the reciprocal social interaction of freely moving WT and TLR-2 KO mice with same strain (C57/BL) or different strain mice (CD1 ICR). Both the duration and the number of contact to the C57BL (data not shown) as well as CD1 ICR test mice were significantly lower in TLR-2 KO mice than to WT mice (Fig. 1j, k). However, attacking behavior against the test mice was not observed in either TLR-2 KO or WT mice during this test (data not shown). In addition, to test aggressive behavior against an intruder in TLR-2 KO mice, we conducted the cotton bud biting test, which can directly provoke the test mice. TLR-2 KO mice showed a significant increase in the number of biting attacks and the duration of attacks against a cotton swab compared to WT mice (Fig. 1l). Collectively, these data indicate that TLR-2 KO mice show significant deficits in social behaviors, including impaired social recognition, reduced social interaction, and aggressive behavior.

### Impaired cognitive and sensorimotor gating function in TLR-2 KO mice

**Figure 2:**
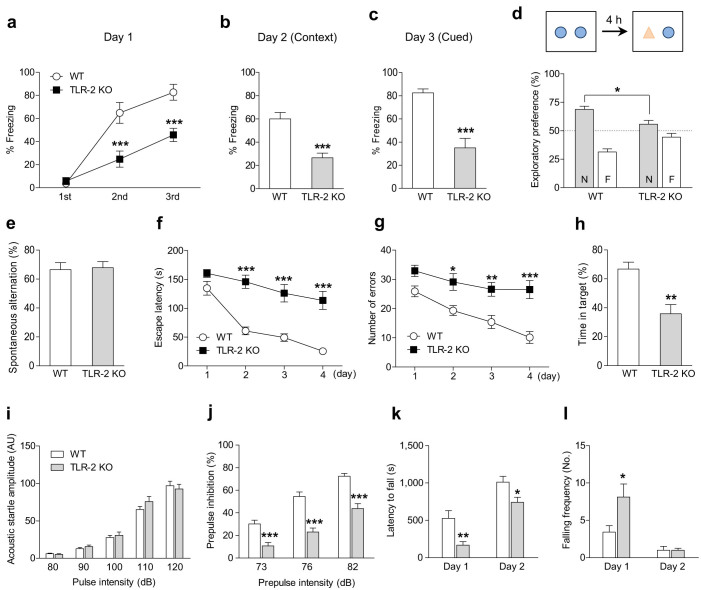
TLR-2 KO mice exhibit cognitive impairments and sensorimotor gating disruption. TLR-2 KO mice (closed squares, *n* = 8) showed a reduced freezing response immediately after the 2nd and 3rd foot shocks (a) as well as in the context test on day 2 (b) and in the cued test on day 3 (c) compared to WT (open circles, *n* = 9) mice in the fear conditioning test. (d) TLR-2 KO mice showed a reduced preference for a novel object after 4 h training in the novel object recognition test compared to WT mice (*n* = 9 per each group). N, novel; F, familiar. (e) Both WT and TLR-2 KO (*n* = 9 per each group) mice had spontaneous alternation in the Y-maze test. Barnes circular maze test (f–h, *n* = 10 per each group). TLR-2 KO mice showed longer times to escape (f) and an increased number of errors to find a platform (g) compared to WT mice. (h) In the probe test, TLR-2 KO mice stayed significantly less time in the target area compared with WT mice. (i, j) To assess sensorimotor gating function, the acoustic startle response test was conducted. (i) No significant differences between WT (*n* = 17) and TLR-2 KO (*n* = 15) mice were observed in acoustic startle amplitude. (j) TLR-2 KO mice showed prepulse inhibition deficits at all prepulse levels compared with WT mice. (k, l) Motor function and coordination were determined using the rota-rod test (WT, *n* = 7; TLR-2 KO, *n* = 8). TLR-2 KO mice showed a decrease in the latency to first falling (k) and an increase in the number of falling frequencies during 20 min (l) when compared to WT mice in the rota-rod test. *P < 0.05, **P < 0.01, ***P < 0.001 vs. WT mice. All data are shown the mean ± s.e.m.

It is well known that schizophrenia patients exhibit broad-based cognitive impairments across a range of cognitive abilities, such as attention, speed of processing, working and long-term memory, executive function, and social cognition^1^. Therefore, we attempted to assess the level of cognitive abilities in WT and TLR-2 KO mice using various behavioral tests. On day 1 of the fear conditioning test, TLR-2 KO mice showed a significantly reduced freezing response immediately after the 2nd and 3rd foot shocks compared to WT mice (Fig. 2a). During the test, we did not observe any difference of pain sensitivity between TLR-2 KO and WT mice. In addition, the impairment in the motor control of freezing or reduction in shock sensitivity compared to WT mice was not observed in TLR-2 KO mice (data not shown). TLR-2 KO mice also showed a significantly reduced freezing behavior both in the context test on day 2 (Fig. 2b) and in the cued test on day 3 (Fig. 2c) compared to WT mice, suggesting that the attention and conditioning memory processes may be disrupted in TLR-2 KO mice. In the novel object recognition test, the exploration time for a new object at 4 h after training was significantly reduced in TLR-2 KO mice compared to WT mice (Fig. 2d), suggesting that the recognition memory may also be impaired in TLR-2 KO mice. However, both WT and TLR-2 KO mice showed similar exploratory activity and preference for the two identical objects in the training phase (Supplementary Fig. 5a and b). In short-term memory performance using the Y-maze test, however, both WT and TLR-2 KO mice had similar spontaneous alternations (Fig. 2e), suggesting that short-term memory may not be impaired in TLR-2 KO mice. To assess spatial working learning and memory, we performed the Barnes circular maze test, which is commonly used to evaluate learning ability and the acquisition of spatial memory. TLR-2 KO mice took a longer time to escape (Fig. 2f) and showed a higher number of errors (Fig. 2g) than WT mice during the training trials, which indicate that the learning ability and the acquisition of spatial memory may be impaired in TLR-2 KO mice. In the probe test, the time spent in the target zone was significantly shorter in TLR-2 KO mice compared to WT mice (Fig. 2h). However, there was no difference in the distance traveled between WT and TLR-2 KO mice (data not shown). Thus, our data suggest that spatial learning and memory may be impaired in TLR-2 KO mice.

Schizophrenia patients show a loss of sensorimotor gating function, which is reflected in a reduced prepulse inhibition (PPI) of the startle reflex as well as deficits in motor coordination^21^. To assess sensorimotor gating function in TLR-2 KO mice, we performed an acoustic startle response test. Two-way ANOVA analysis revealed that there were no significant differences in the acoustic startle response between TLR-2 KO and WT mice (Fig. 2i), suggesting that hearing ability may be not impaired in TLR-2 KO mice. Although PPI monotonically increased with increased prepulse intensities in all genotypes, TLR-2 KO mice showed a significantly lower PPI level than WT mice at all prepulse levels (Fig. 2j). In the rota-rod test, TLR-2 KO mice also showed faster falling on the rod (Fig. 2k) and an increased number of falls than WT mice on the first day (Fig. 2l), indicating that TLR-2 KO mice may have deficits in motor coordination. Taken together, these results suggest that TLR-2 KO mice exhibit PPI deficits likely due to sensorimotor gating dysfunction and motor incoordination.

Several lines of evidences have suggested that the deficits in learning and memory in schizophrenia may be mediated through disrupted synaptic plasticity including long-term potentiation (LTP) or long-term depression (LTD)^22^. Thus, we characterized the neural plasticity of LTP in hippocampal slice cultures as well as LTD in cerebellar slice cultures of WT and TLR-2 KO mice. LTP at hippocampal CA3-CA1 synapses was not altered (Supplementary Fig. 6a), whereas cerebellar LTD was inhibited in TLR-2 KO mice compared to WT mice (Supplementary Fig. 6b). It was reported that an impairment of parallel fiber LTD is sufficient to disrupt the encoding and storage of cerebellum-dependent motor memory^23^. Collectively, our data suggest the possibility that deficits in the wide ranges of the learning and memory system, including attention, conditioning, recognition, and spatial memories, in TLR-2 KO mice may be, at the very least, associated with the disrupted cerebellar LTD.

### TLR-2 KO mice show ventricle enlargement and higher cell death than WT

**Figure 3:**
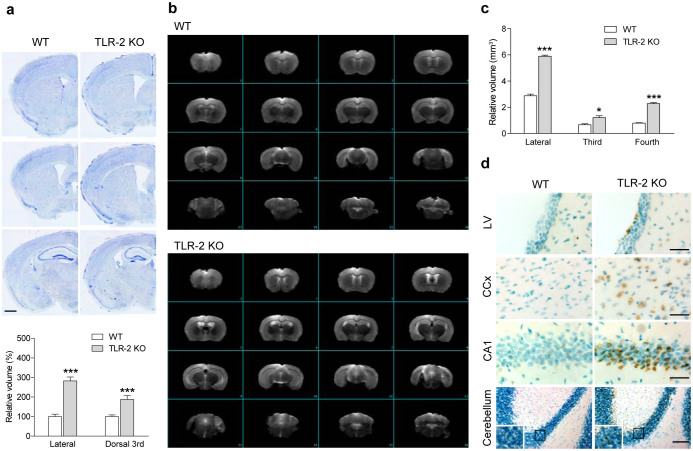
TLR-2 KO mice exhibit ventricle enlargement and more cell death than in WT mice. (a) Representative photograph (top) and volumetric analysis (bottom) of WT (four slices each from six animals) and TLR-2 KO (four slices each from seven animals) mouse brains stained with Nissl bodies. Both lateral and dorsal 3rd ventricle enlargements were observed in the brains of adult TLR-2 KO mice compared to WT mice. The values for ventricle volumes in TLR-2 KO mice are indicated as the percentages of the WT values. Scale bar, 1,000 μm. (b) MRI images of WT (top) and TLR-2 KO (bottom) mouse brains. MRI images were obtained using a 9.4 T/160 mm animal MRI system. Volumetric analyses of MRI images were performed using the VnmrJ 4.0 software provided with the scanner (Agilent Technologies) according to the adult mouse brain atlas. (c) The volumes of lateral, third, and fourth ventricles were significantly larger in TLR-2 KO mice compared with WT mice (*n* = 3 per each group). All data are shown the mean ± s.e.m. *P < 0.05, ***P < 0.001 vs. WT mice. (d) TUNEL revealed that higher numbers of apoptotic cell death were observed in the ependymal layer of the lateral ventricle (LV), cerebral cortex (CCx), CA1 region of hippocampus (CA1), and cerebellum in the TLR-2 KO mice compared to WT mice. The rectangular subsets were magnified in the rectangle region in each photomicrograph. Bar, 30 μm in the LV, CCX, or CA1; Bar, 100 μm in the cerebellum.

Neuroimaging and postmortem studies have shown that ventricular enlargement has been observed in the schizophrenic brain, which have significant correlations with the severity of symptoms of schizophrenia^2^,^24^. To examine whether TLR-2 KO mice would show the morphological characteristics of schizophrenia including ventricle enlargement, we performed histochemical and magnetic resonance imaging (MRI) studies. Nissl staining revealed that both lateral and dorsal 3rd ventricles were significantly enlarged in the brains of adult TLR-2 KO mice compared to WT mice (Fig. 3a). MRI data also showed that the volume of ventricles including the lateral, third, and fourth were significantly larger in TLR-2 KO mice than WT mice (Fig. 3b, c). Because the number of cell deaths may contribute these morphological changes in the mouse brain, we examined whether progressive cell death occurred in TLR-2 KO mice. TUNEL assay revealed that a higher cell death was observed in the ependymal layer of the lateral ventricle, cerebral cortex, CA1 region of hippocampus, and cerebellum in the TLR-2 KO mice than in WT mice (Fig. 3d). In addition, the intensity of Iba-1^+^ or GFAP^+^-glial cells in the hippocampal region were higher in TLR-2 KO mice than WT mice (Supplementary Fig. 7), which imply abnormal activation of glial cells in TLR-2 KO mice. Thus, these data provide the possibility that the higher cell death in the various brain regions, including the ependymal layer of the ventricles and abnormal activation of glial cells may be linked to the ventricle enlargement in TLR-2 KO mice.

### Alteration of Akt-GSK-3 signaling in TLR-2 KO mice

**Figure 4:**
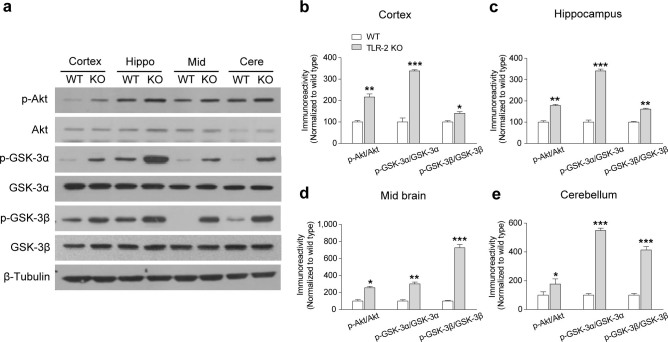
The levels of phosphorylated Akt and GSK-3 are spontaneously increased in TLR-2 KO mice. (a) Immunoblots of selected brain regions, including cerebral cortex, hippocampus (Hippo), midbrain (Mid), and cerebellum (Cere), of WT and TLR-2 KO mice. Quantitative analysis of immunoblots showed that the levels of p-Akt, p-GSK-3α, and p-GSK-3β were significantly higher in the cerebral cortex (b), hippocampus (c), midbrain (d), or cerebellum (e) region of TLR-2 KO mice compared to WT mice (*n* = 3 per each group). The gels have been run under the same experimental conditions. All data are shown the mean ± s.e.m. *P < 0.05, **P < 0.01, ***P < 0.001 vs. WT mice.

Recent evidence suggests that an altered Akt-GSK-3 signaling cascade is closely associated with the pathogenesis of schizophrenia as well as the psychotomimetic effects in the animal models of schizophrenia^18^. Moreover, it has been implicated that TLR-2 dependent signaling is directly linked to the phosphoinositide-3-kinase (PI3K)-Akt signaling pathway^25^. Therefore, we examined whether Akt and GSK-3 activities were altered in TLR-2 KO mice. By Western blot analysis, we found that the levels of phosphorylated Akt (p-Akt), phosphorylated GSK-3α (p-GSK-3α), and phosphorylated GSK-3β (p-GSK-3β) were significantly higher in the cortex, hippocampus, midbrain, or cerebellum region of TLR-2 KO mice than WT mice (Fig. 4a–e). However, the total protein levels of Akt, GSK-3α, or GSK-3β were not different between TLR-2 KO and WT mice. Thus, these data indicate that TLR-2 KO mice show altered Akt and GSK-3 activities in the brain.

### Anti-psychotics attenuate the PPI deficits and reduce Akt-GSK3 activation in TLR-2 KO mice

**Figure 5:**
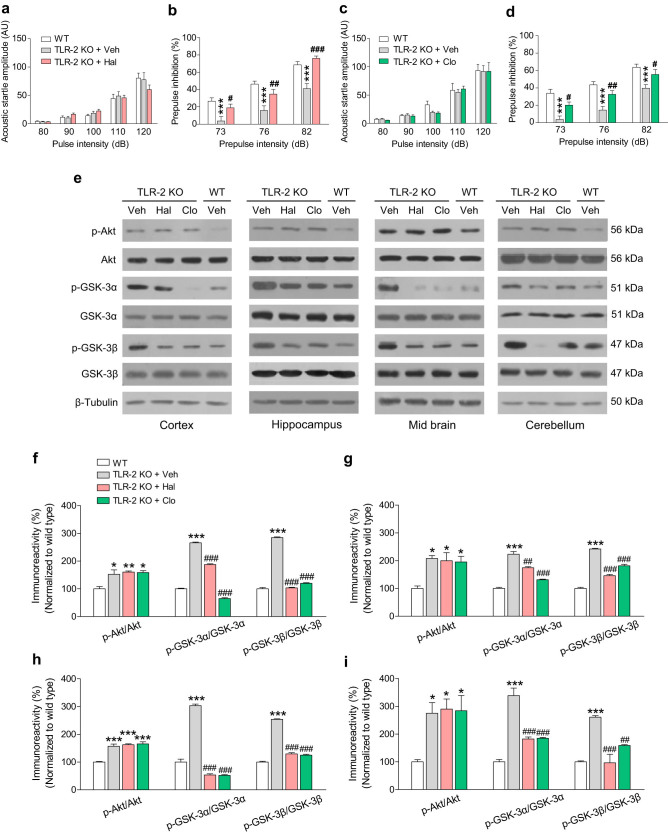
Anti-psychotic drugs attenuated the PPI deficits and activation of Akt-GSK3 signaling in TLR-2 KO mice. In the acoustic startle response test, acute treatment with haloperidol (Hal, a) or clozapine (Clo, c) did not affect the level of acoustic startle amplitude in TLR-2 KO mice. However, PPI deficits in TLR-2 KO mice were significantly attenuated by the acute administration of haloperidol (b) or clozapine (d), respectively. (e) Immunoblots of p-Akt, p-GSK-3α, and p-GSK-3β were shown in the selected brain regions of TLR-2 KO and WT mouse brains 1 h after the administration of haloperidol or clozapine. The increased expression level of p-GSK-3α and p-GSK-3β, but not p-Akt, was significantly decreased by antipsychotics in the cortex (f), hippocampus (g), midbrain (h), and cerebellum (i) region of TLR-2 KO mice (*n* = 3 per each group). The gels have been run under the same experimental conditions. All data are shown the mean ± s.e.m. **P < 0.01, ***P < 0.001 vs. WT mice; ^#^*P* < 0.05, ^##^*P* < 0.01, ^###^*P* < 0.001 vs. TLR-2 KO mice. Veh, vehicle.

To investigate whether PPI deficits in TLR-2 KO mice can be reversed by typical (haloperidol) or atypical (clozapine) antipsychotics, we conducted the acoustic startle response test. In line with Figure 2i–j, PPI deficits were observed in TLR-2 KO mice, although the acoustic startle response was unchanged (Fig. 5a–d). As expected, the PPI deficits in TLR-2 KO mice were significantly rescued by the acute administration of haloperidol (1 mg per kg body weight) or clozapine (1 mg per kg body weight) (Fig. 5b, d). However, acute treatment with haloperidol or clozapine did not affect the level of acoustic startle response in TLR-2 KO mice (Fig. 5a, c). In addition, acute treatment with haloperidol or clozapine did not affect the level of acoustic startle amplitude and PPI in WT mice (Supplementary Fig. 8a, b). These results are in agreement with the previous reports that PPI deficits could be attenuated by antipsychotics in animal models or clinical cases of schizophrenia^26^.

Next, because abnormalities in Akt-GSK-3 signaling have been shown to be closely associated with sensorimotor gating function^27^, we evaluated whether the increased levels of p-Akt or p-GSK-3α/β in TLR-2 KO mice would be also reduced by treatment with such antipsychotics. Western blot analysis revealed that the increased expression level of p-GSK-3α/β, but not p-Akt, was markedly reduced in the cortex, hippocampus, midbrain, and cerebellum region of TLR-2 KO mice when treated with haloperidol or clozapine (Fig. 5e–i). However, the expression levels of p-Akt, p-GSK-3α, and p-GSK-3β in the cortex, hippocampus, midbrain, or cerebellum region of WT mice were not changed by the acute administration of haloperidol or clozapine compared to the vehicle-treated controls (Supplementary Fig. 8c). These results suggest that PPI deficits of sensorimotor gating function in TLR-2 KO mice may be associated with the alteration in GSK-3α/β activities.

### Direct linkages between TLR-2 and Akt-GSK-3 signaling

**Figure 6:**
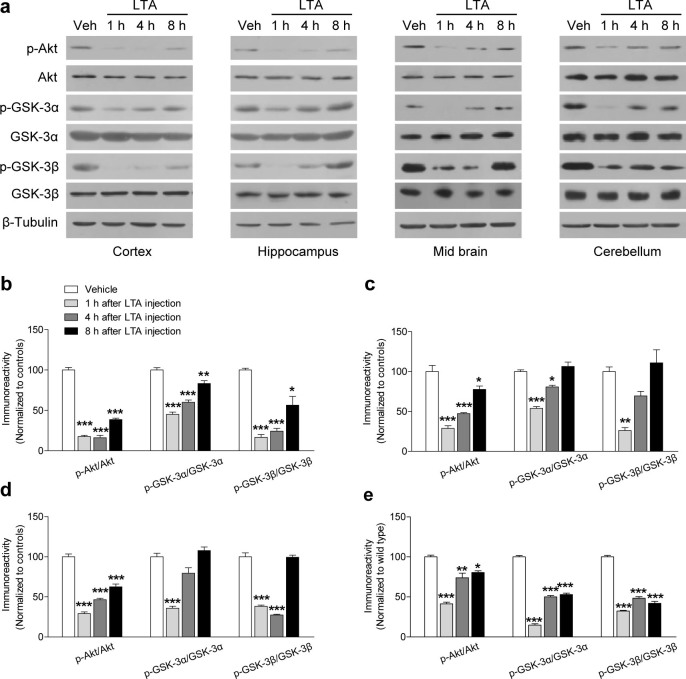
Direct linkages between TLR-2 and Akt-GSK-3 signaling. To investigate whether the activation of TLR-2 by lipoteichoic acid (LTA), a TLR-2 ligand, changes the level of p-Akt, p-GSK-3α, and p-GSK-3β in WT mice, each brain region including cerebral cortex, hippocampus, midbrain, and cerebellum of WT mice was isolated at the indicated time (1 h, 4 h, or 8 h, respectively) after administration of LTA (50 μg, i.p.). Immunoblots (a) and its quantitative analysis (b–e) were represented the selected brain regions of WT mouse brains after administration of LTA. The expression levels of p-Akt, p-GSK-3α, or p-GSK-3β were decreased at 1 h after the acute administration of LTA, and time-dependently recovered to control levels in the cortex (b), hippocampus (c), midbrain (d), or cerebellum (e) region compared to the vehicle-treated controls (*n* = 3 per each group). The gels have been run under the same experimental conditions. All data are shown the mean ± s.e.m. *P < 0.05, **P < 0.01, ***P < 0.001 vs. WT mice. Veh, vehicle.

To clarify the direct linkage between TLR-2 and Akt-GSK-3 signaling, we further investigated whether the systemic injection of LTA, a TLR-2 ligand, would affect Akt-GSK signaling in WT and TLR-2 KO mice. In WT mice, Western blot analysis revealed that the levels of p-Akt or p-GSK-3α/β were decreased at 1 h after acute administration of LTA (50 μg, i.p.) in the cortex, hippocampus, midbrain, or cerebellum region when compared to vehicle-treated controls and, thereafter, recovered to basal level (Fig. 6a–e). However, the increased levels of p-Akt (Supplementary Fig. 9) or p-GSK-3α/β (data not shown) in the cortex, hippocampus, midbrain, or cerebellum region of TLR-2 KO mice were not changed by LTA. These results suggest that TLR-2 signaling may be closely linked to the Akt-GSK-3 signaling pathway.

## Discussion

Until now, most studies on the roles of TLRs have been focused on the immune system and the mechanisms underlying pathological conditions, such as pathogen recognition and the initiation of inflammatory and immune responses that trigger both innate and adaptive immune responses^28^,^29^. In the central nervous system (CNS), TLRs have also been known to play important roles in the pathogenesis of brain diseases, such as stroke, Alzheimer's disease, and multiple sclerosis^13^,^30^. Recently, emerging evidences have shown the novel roles of TLRs in the CNS without any immune challenge, such as the regulation of neurodevelopment, adult neurogenesis, neuronal plasticity, mood-related behaviors, and learning and memory system^12^,^13^,^14^,^15^,^31^,^32^. Notably, a multi-stage schizophrenia genome-wide association study provides support for the speculated link between the immune system and schizophrenia^33^. In the present study, we first demonstrated that a deficiency of the *TLR-2* gene caused schizophrenia-like behavioral, histological, and biochemical characteristics in mice. Importantly, several antipsychotics were effective in mitigating the disruption of sensorimotor gating in TLR-2 KO mice. Moreover, TLR-2 KO mice exhibited morphological changes, including ventricle enlargements, which are relevant to brains of schizophrenia^24^,^34^. However, it should be pointed out that this study is limited to provide the direct evidence which TLR-2 is associated with schizophrenia. Schizophrenia is a complex multigenic disorder whose etiology can vary between ethnicities and subpopulations. Loss of a single gene in a mouse model does not replicate all aspects of schizophrenia. For example, Muller et al. reported that TLR-2 was not elevated in the blood samples of schizophrenia patients compared to healthy controls^35^. On the other hand, the *TLR-2* gene is located at chromosome 4q32, a region that has been shown to be linked to positive schizophrenia-interpersonal sensitivity in schizophrenia patients^36^. A significant association between *TLR-2* gene polymorphisms and clinical cognitive symptoms has also been observed in schizophrenia patients^37^. Thus, these reports demonstrate that TLR-2 changes may not be present in all populations of schizophrenia. Nevertheless, it would be noteworthy that our study provides first evidences on the correlation of innate immune system and psychiatric disorders including schizophrenia.

We found that TLR-2 KO mice exhibited hyperactivity during exposure to the open field. It is well known that increased locomotion or stereotype behaviors are observed in a subset of schizophrenia patients, as well as in animal models elicited by pharmacological and genetic approaches^38^,^39^. Thus, the persistent locomotor hyperactivity in TLR-2 KO mice may mimic the positive symptom of “psychomotor agitation” in schizophrenic patients^40^. In addition to hyperlocomotion, TLR-2 KO mice showed reduced anxiety-like behaviors, but not a depression-like behavior. A similar study showed that TLR-3 deficient mice exhibit an impaired amygdala-related anxiety behavior, suggesting the possibility that TLR signaling may be normally involved in amygdala plasticity^12^. Given that anxiety and depressive symptoms are frequently observed in the course of schizophrenia^41^, therefore, our data suggested that TLR-2 mediated signaling is likely not to relevant to the mood-related symptoms of the illness. Additionally, negative symptoms of schizophrenia are characterized as social withdrawal, avolition, and anhedonia, which are extremely difficult to replicate in animal models^16^,^40^. Nevertheless, deficits in social functions, e.g., social interaction, social cognition, and aggression, can be represented in both human and animal cases^42^. By social behavioral tests, we found that TLR-2 KO mice showed a tendency to avoid stranger mouse and an impaired social recognition. Furthermore, TLR-2 KO mice responded to the direct stimulation by the cotton bud, which led to provoking the mice and inciting the aggressive behavior. Taken together, TLR-2 KO mice exhibited persistent hyper-locomotion behavior and abnormal social behavior, which are similar to the positive and negative symptoms of schizophrenia^20^. Notably, the aggressive behaviors began to be observed from 8 weeks old, but were not presented in younger mice, which is similar to the effects of age in some cases of schizophrenia in humans^1^.

PPI is widely regarded as an endophenotypic marker for sensorimotor gating in schizophrenia and is sensitive to treatments generating hyperdopaminergic and hypoglutamatergic states^43^. Although several other neuropsychiatric disorders, including obsessive-compulsive disorder, autism spectrum disorder, and bipolar disorder, are also associated with decreased PPI^21^,^44^,^45^, PPI deficit is the best characterized and the most widely replicated characteristic in the schizophrenia patients^46^. To our knowledge, we are the first to observe the disruption in sensorimotor gating in TLR-2 KO mice as measured by PPI. PPI deficits in TLR-2 KO mice were also reversed by an acute administration of typical or atypical antipsychotics. In addition, various cognitive tests revealed that broad learning and memory systems were impaired in TLR-2 KO mice, which may be due to sensorimotor gating dysfunction. Moreover, TLR-2 KO mice also showed a deficit in motor coordination in the rota-rod test. Recent evidence has shown that motor abnormalities and cognitive impairments in schizophrenia are closely related with the abnormalities in the structure and function of the cerebellum^47^,^48^. Furthermore, we observed that cerebellar LTD was impaired in TLR-2 KO mice. Thus, these results suggest the possibility that TLR-2 signaling may be involved in cerebellum plasticity.

Based on morphological studies, ventricle enlargement was observed in the brains of TLR-2 KO mice compared to WT mice. Furthermore, compared to WT mice, TLR-2 KO mice showed higher numbers of apoptotic cell deaths in the cerebral cortex, hippocampus, cerebellum, and the ependymal layer of the ventricles as well as the activation of glial cells, including astrocyte and microglia, in the hippocampus in the absence of apparent tissue abnormality. It has been demonstrated that differentiation of neural progenitor cell into neurons is disrupted in TLR-2 KO mice, leading to increased glial cell numbers rather than neurons^14^, similar with our observation. Therefore, these results indicate that TLR-2 may play an important role in neuronal cell fate and the development of the ventricular system^32^. Moreover, several lines of evidence show that abnormal adult neurogenesis is closely associated with not only disruption in sensorimotor gating (PPI), mood, and memory function, but also the pathogenesis of psychiatric disorders such as schizophrenia^49^,^50^,^51^. Given that PPI deficits and impaired broad learning and memory system were presented in TLR-2 KO mice, impaired hippocampal neurogenesis in TLR-2 KO mice may contribute to sensorimotor gating dysfunction, resulting in PPI deficits and cognitive impairment.

The innate immune initiation by TLR-2 is known to be mediated by MyD88-dependent NF-κB activation. In contrast to our results from TLR-2 KO mice, it has been reported that the Myd88 deficiency in C57BL/6 mice showed more anxiety, better motor coordination, and improved spatial learning than WT mice^52^. On the other hand, recent studies have shown that the Akt-GSK-3 signaling pathway is closely associated with the function of TLRs in the immune system. The stimulation of TLR-2 is known to regulate Akt activity through Mal/PI3K signaling cascade^25^, which directly activates NF-κB, an orchestrator of the inflammatory response^53^. However, it is still unclear whether deletion of TLR-2 gene also affects the Akt activity and its downstream signaling cascade, especially in the brain. In the present study, we first observed that the activities of Akt and GSK-3α/β were markedly changed in the brains of TLR-2 KO mice. Moreover, the activation of TLR-2 by LTA caused the dephosphorylation of Akt and GSK-3α/β in WT mice, but failed to show those effects in TLR-2 KO mice. These data indicate that the Akt-GSK-3 signaling pathway may be directly linked to TLR-2 signaling in both WT and TLR-2 KO mice. However, the mechanism underlying how *TLR-2* gene deletion changes Akt activity is unclear, and further study would be required to clarify these issues. In addition, PPI deficits as well as the inhibition of GSK-3α/β in TLR-2 KO mice were attenuated by both clozapine and haloperidol. It is well documented that the Akt-GSK-3 signaling pathway plays crucial roles in mediating diverse neuronal processes including brain development, regulation of synaptic plasticity, and determination of cell fate^54^. Moreover, dysregulation of the Akt-GSK-3 signaling pathway is associated with the pathogenesis of numerous neurological and psychiatric disorders, including schizophrenia^17^,^18^,^54^. Collectively, these findings suggest that our observations, such as behavioral alterations (at least sensorimotor gating disruption), ventricle enlargement, and abnormal synaptic plasticity, in TLR-2 KO mice may be linked in part to the Akt/GSK-3 signaling cascade. Additionally, Emaminan et al. showed a decrease in Akt1 protein levels and, consequently, a reduced phosphorylation activity of GSK-3β in the peripheral lymphocytes and brains of individuals with schizophrenia^18^. They also showed that Akt deficient mice did not exhibit any change in PPI level compared with WT mice. On the contrary, we observed a significant increase in the phosphorylation levels of Akt and GSK-3α/β, but not in the level of total form of Akt or GSK-3, in the brains of TLR-2 KO mice compared to WT mice. It would be notable that acute administration of psychostimulants, such as DA receptor agonists, 5-HT receptor agonists, and NMDA receptor antagonists, induces phosphorylation of Akt or GSK-3β in the frontal cortex, which may lead to psychotomimetic activity such as sensorimotor gating disruption and repetitive movements in mice^55^. The GSK3 inhibitor, including SB216763 and linopirdine, reduces PPI when directly infused into the medial prefrontal cortex in mice^27^. Thus, the role of Akt-GSK-3 signaling in psychotic symptoms such as PPI may be different in rodents and in humans, especially in schizophrenia. Additional works would be required to resolve these issues. In addition, Emaminan et al. showed that chronic administration of haloperidol (for 12 days) increased the phosphorylation of Akt and GSK-3β in the C57Bl/6 mice brain, but acute treatment with haloperidol (at 2 h) did not affect the Akt phosphorylation of Ser 473^18^. In this study, we did not find any significant changes in the phosphorylation of Akt (Ser 473) and GSK-3α/β at 1 h after haloperidol treatment. The discrepancy in the effect of haloperidol may be attributable to the injection paradigm (1 h-acute versus 12 days-chronic).

In conclusion, we have conducted comprehensive behavioral, biochemical, and morphological analyses of TLR-2 KO mice. Behavioral alterations in TLR-2 KO mice were relevant to the symptoms of schizophrenia, such as the positive, negative, and cognitive symptoms. In addition, ventricles enlargements, apoptotic cell death, and marked dysregulation of Akt-GSK-3 signaling were also observed in the brain of TLR-2 KO mice. Although the critical issue of whether prenatal immune activation could induce schizophrenia-like symptoms should be investigated, the present study suggests that an alteration of TLR-2 functions in the CNS may contribute to schizophrenia etiology.

## Methods

### Materials

Lipoteichoic acid (LTA), haloperidol, and clozapine were purchased from Sigma-Aldrich Chemical Co. (St. Louis, MO). Haloperidol was dissolved in saline with 1% dimethyl sulfoxide. Clozapine was dissolved in saline with 0.1 M hydrochloric acid. LTA were dissolved in saline. Antibodies to GSK-3α, GSK-3β, Akt, and β-actin were purchased from Santa Cruz Biotechnology, Inc. (Santa Cruz, CA). Antibodies to p-Akt at serine 473, p-GSK-3β at serine 9, and p-GSK-3α at serine 21 were purchased from Cell Signaling Technology (Danvers, MA). All other materials consisted of the highest grades available and were obtained from typical commercial sources.

### Animals

TLR-2 KO mice backgrounds from C57BL/6 mice were obtained from Dr. Sung J. Lee (Seoul National University, Seoul, Korea)^56^. C57BL/6J mice used as wild-type (WT) control and CD1 ICR mice used for the reciprocal social interaction test were purchased from Daehan BioLink Inc. (Eumseong, Korea). Mice (8–20 weeks old) used for all behavioral tests were performed blind to genotypes with age-matched littermate pairs of mice. The mouse cages were maintained at a constant temperature (23 ± 1°C) and relative humidity (60 ± 10%) under a 12-h light/dark cycle (lights on from 07:30 to 19:30), and the facilities were approved by the Association for Assessment and Accreditation of Laboratory Animal Care International (AAALAC-i). Animals were allowed access to water and food *ad libitum*. Animal maintenance and treatment were carried out in accordance with the Animal Care and Use Guidelines issued by Kyung Hee University, Korea. All experiments with mice were performed according to the protocols approved by the Institutional Animal Care and Use Committee of Kyung Hee University (Approved protocol No. KHP-2010-12-11). The number of mice used in each experiment is stated in Supplementary Table 1. The naive mice were used for sampling of biochemical or physiological studies.

### Tests for mood-related behaviors

#### Open field test

To determine the spontaneous horizontal locomotor activity of WT and TLR-2 KO mice, an open-field test was performed as described below. The test was carried out in clear black Plexiglas boxes (41.5 cm × 41.5 cm × 41.5 cm) equipped with the video-based Ethovision System (Noldus, Wageningen, Netherlands). Mice were placed in the center of the apparatus and locomotor behaviors were recorded for 1 h using a video-tracking system. Horizontal locomotor activity was expressed as total ambulatory distance. The number of animals that visited the center zone (15 cm × 15 cm) was recorded. The test box was cleaned with 70% ethanol between each test.

#### Elevated plus-maze test

To evaluate the anxiety-like behavior of WT and TLR-2 KO mice, an elevated plus-maze test was conducted as described below. The mouse was placed at the center of the maze platform with its head facing an open arm. Animals were tested individually, once only, for 5 min, and the maze was cleaned after each trial to remove any residue or odors. The number of entries into the open and closed arms, the time spent in each arm, and the total distance moved was measured and analyzed using the video-based Ethovision System (Noldus, Wageningen, Netherlands).

#### Marble-burying test

To assess the anxiety-like behavior of WT and TLR-2 KO mice, a marble-burying test was conducted as described below. The test was conducted in a new cage (equally sized and illuminated as the home cage) with 20 clear glass marbles (20 mm diameter) evenly spaced in 6 cm depth of sawdust^57^. During the test, the mice had access to food and water, and the test cage was covered with a metal grid. After 30 min, the number of marbles that were buried in more than two-thirds of sawdust was counted.

#### Forced swimming test

To assess the depressive-like behavior of WT and TLR-2 KO mice, a forced swimming test was performed as described below. A mouse was placed in a 25 cm glass cylinder (14 cm diameter) containing a 20 cm height of water maintained at 24 ± 2°C; the mouse was forced to swim for 6 min. The immobility duration was recorded using a video-based Ethovision System (Noldus, Wageningen, Netherlands) during 6 min.

### Tests for social behavior

#### Three-chamber social novelty preference test

To evaluate the social function of WT and TLR-2 KO mice, the three-chamber social novelty preference test was conducted as described in a previous study with minor modifications^58^. The setup consisted of a rectangular Plexiglas box (w × d × h: 52 × 25 × 23 cm) divided into 3 chambers. In the habituation trial (5 min), empty wire cages (height × diameter: 12 × 8 cm) that can provide contact with another mouse were present in the left and right chambers visible from the middle chamber. In the sociability trial (10 min), a novel mouse from same strain that has never been observed before was placed in the wire cage of the left chamber, whereas no mouse was placed in the wire cage of the right chamber. Immediately after the sociability trial, the social recognition trial (10 min) was conducted wherein another novel mouse was placed in the wire cage of the right chamber. Each trial, the test mouse was placed in the central chamber and explored all three chambers. The time spent in each chamber was recorded. At the end of each test, the apparatus and wire cages were cleaned with paper towels using 70% ethanol.

#### Reciprocal social interaction test

To evaluate the social interaction of WT and TLR-2 KO mice, the reciprocal social interaction test was performed as described below. The test mouse and an age- and gender-matched stimulus mouse (CD1 ICR or C57/BL) were introduced in a neutral cage with fresh bedding. The cage was used only once. The mice had not interacted previously with other mice. The social interactions between mice were recorded by video camera and PC-based video capture software for 10 min. The time spent in aggressive interactions, such as attacking, wrestling, and biting the dorsal surface, and the time spent in non-aggressive interactions, such as nose-to-nose sniffing, anogenital sniffing, and grooming, were measured manually using the event-recording function in the video-tracking software by a researcher who was blind to the genotype of test mice.

#### Cotton bud biting test

To test the aggressive behavior of WT and TLR-2 KO mice, the mice were held with the hand without pressing on the trachea, and a sterilized cotton bud was put to the face of the mice. Aggressive behavior was characterized by a violent biting attack against the cotton bud. The mice were given 10 trials. Analysis was performed by counting the total number of biting attacks and the average duration of biting. Five second was set as the maximum time of biting (cut-off time).

### Tests for learning and memory

#### Fear conditioning test

To assess the conditioning memory of WT and TLR-2 KO mice, a fear-conditioning test was performed as previously described^59^. All training and testing took place in two modular test chambers. The two chambers differed in several aspects: chamber A (13 cm × 14 cm × 30 cm) was a square black acryl box, whereas chamber B (13 cm × 16 cm × 30 cm) was a rectangular plain acryl box. The two chambers were kept in different rooms and cleaned with different substances. Each chamber was located inside a larger, insulated plastic cabinet that provided protection from outside light and noise. The behavior of mice was recorded by digital video cameras mounted above the conditioning chamber. To assess freezing, distal videos were scored by two observers using a stopwatch, and the average of freezing time was analyzed. Freezing was defined as the complete absence of motion, including the motion of the vibrissae, for a minimum of 0.5 s. Each test session was scored continuously in its entirety. The fear conditioning procedure was conducted over 3 days. On day 1, animals were individually placed in the conditioning chamber A and allowed to freely explore for 90 s. The conditioned stimulus (CS) was a 30 s tone of 90 dB, and the unconditioned stimulus (US) was a 0.7 mA electric foot-shock for 1 s delivered at the end of the CS from the floor bar in chamber A. CS-US paring trials were delivered 3 times, and the inter-trial interval was 60 s and 120 s, respectively. The chambers were cleaned with 70% ethanol between each set of mice. Each chamber was scented by a paper towel dabbed with mint solution and placed underneath the chamber floor. On day 2, mice were tested for conditioned fear of the training context. Animals were individually placed in the conditioning chamber A. The testing procedure and context were identical to those used on day 1, except the CS and US were not presented. Mice were placed into the chambers for 10 min. The freezing was scored for the entire session. On day 3, the procedure and context were changed in several ways to test the conditioned fear of the tone, the CS, in the absence of contextual cues associated with shock. Animals were individually placed in conditioning chamber B; the chamber was scented with 0.3% acetic acid; the ventilation fan was not operated; the experimenter wore a different style of gloves; the chambers were cleaned with a non-alcohol disinfectant between runs; and the mice were kept in a different holding room before testing. The tone was presented once for 30 s into the session. No shocks were administered. The freezing time was measured for the entire session.

#### Novel object recognition test

To assess the object recognition memory of WT and TLR-2 KO mice, the novel object recognition task was carried out as previously described^60^. The experimental apparatus consisted of a black polyvinyl plastic square open field (25 cm × 25 cm × 25 cm). Habituation training was conducted by exposing the animal to the experimental apparatus for 5 min per day in the absence of objects for 2 days. During the training session, mice were placed in the experimental apparatus in the presence of two identical objects and allowed to explore for 5 min. After a retention interval of 4 h, mice were again placed in the apparatus; however, one of the objects was replaced with a novel object. Mice were also allowed to explore for 5 min. The objects chosen for this experiment included a metal cylinder and a plastic rectangular block, both approximately the same height. The durations of time mice spent exploring each object (familiar object, *T_familiar_*; novel object, *T_novel_*) were recorded.

#### Y-maze test

To assess the working memory of WT and TLR-2 KO mice, a Y-maze test was conducted in a three-arm maze with angles of 120° between the arms; the arms were 40 cm long and 3 cm wide with walls that were each 12 cm high. The maze floor and walls were constructed from dark opaque polyvinyl plastic as previously described^61^. Mice were initially placed within one arm, and the sequence and number of arm entries were recorded manually for each mouse over an 8 min period. The percentage of triads in which all three arms were represented, i.e., ABC, CAB, or BCA but not BAB, was recorded as an ‘alternation’ to estimate short-term memory. Arms were cleaned with 70% ethanol between each test to remove odors and residues. The alternation score (%) for each mouse was defined as the ratio of the actual number of alternations to the possible number (defined as the total number of arm entries minus two) multiplied by 100 as shown by the following equation: % Alternation = (Number of alternations)/(Total arm entries - 2) × 100. The number of arm entries was used as an indicator of locomotor activity.

#### Barnes circular maze test

To assess the spatial learning and memory of WT and TLR-2 KO mice, a Barnes circular maze test was performed as described below. The Barnes circular maze is a planar, round white Plexiglas platform (90 cm diameter), 1 m above the floor, with 20 evenly spaced holes (7 cm diameter) located 5 cm from the perimeter. A black escape box (15 × 7 × 7 cm) was placed under one hole. Spatial cues with distinct patterns and shapes were placed on the wall of the testing room. A 60 w light was turned on during the trial. An experimenter remained in the same place with minimal movement throughout the trials. The platform and the escape box were cleaned thoroughly with 70% ethanol and paper towels between each trial to remove olfactory cues. One day before the training trials began, test mice were habituated in the target box for 3 min. The training trials were repeated for 4 consecutive days, and 3 trials per day were performed with 20 min inter-trial intervals. At the beginning of each trial, the mouse was placed in a cylindrical holding chamber (10 cm diameter) located in the center of the maze. After 10 s of holding time, the mouse was allowed to search for the target hole for 3 min. If the mouse failed to find the target hole in 3 min, it was gently guided into the target hole by the experimenter's hands. When the mouse entered the escape box, the light was turned off and the mouse remained undisturbed for 1 min. The movement of the mouse was recorded, and the number of errors made and the latency to find the target hole were measured during the training trials by a video tracking software (Nodulus, Wageningen, Netherlands). On day 5, the probe trial was performed with each mouse. The escape box was removed, and the test mouse was allowed to find the target hole freely for 90 s. During the probe trial, the % time in the target area, the total distance travelled and the latency to find the target hole were measured using the video tracking software.

### Tests for sensorimotor gating and motor coordination

#### Acoustic startle response and prepulse inhibition procedure

To assess the sensorimotor gating function of WT and TLR-2 KO mice, acoustic startle response (ASR) and PPI testing were performed in SR-LAB startle chambers (San Diego Instruments, San Diego, CA) as previously described^60^. In brief, each chamber consisted of a clear non-restrictive Plexiglas cylinder resting on a platform inside a ventilated box. All ASR test sessions consisted of startle trials (pulse alone) and nostimulus trials (NS). The pulse alone trial consisted of a 40 ms 80, 90, 100, 110, or 120 dB pulse of broad-band noise. After the end of the ASR test sessions, the PPI test sessions were started, and all PPI test sessions consisted of pulse alone, prepulse trials (prepulse + pulse), and NS. The pulse alone trial of the PPI test consisted of a 40 ms 120 dB pulse of broad-band noise. Prepulse + pulse trials consisted of a 20 ms noise prepulse, an 80 ms delay, then a 40 ms 120 dB startle pulse (100 ms onset to onset). The acoustic prepulse intensities were 73, 76, and 82 dB (i.e., 3, 6, and 12 dB above, respectively, the 70 dB background noise). The NS trial consisted of background noise only. There was an average of 21 s (range: 12–30 s) between trials. The amount of PPI was calculated as a percentage score for each acoustic prepulse trial type: % PPI = 100 − (startle response for prepulse + pulse)/(startle response for pulse alone of the PPI test)/100. To investigate whether typical or atypical antipsychotic agents affect sensorimotor gating function in TLR-2 KO mice, haloperidol (1 mg per kg body weight) or clozapine (1 mg per kg body weight) was intraperitoneally administered into mice 1 h before the test.

#### Rota-rod test

The rota-rod test is a useful method to study the integrity of cerebellar function, as well as the lower motor unit function. The mice were trained to stay on the rotating rod (diameter, 7.3 cm). Following the training period, the mice were tested twice using a paradigm where the rotational speed was increased from 4 to 20 rpm during 25 min. The test trials were conducted once per day for 2 days, and the latency to first falling off the rod or the falling frequency during 25 min was recorded.

#### Electrophysiological measurements

To examine hippocampal long-term potentiation (LTP), a block of the hippocampus of WT and TLR-2 KO mouse brain was removed and sectioned in the coronal plane into 400 μm thick slices using a Microsliver (DTK 1000, Ted Pella, Inc. Redding, CA). These slices were rapidly placed in ice-cold, oxygenated (95% O_2_ and 5% CO_2_) low-Ca^2+^/high-Mg^2+^ dissection buffer containing 5 mM KCl, 1.23 mM NaH_2_PO_4_, 26 mM NaHCO_3_, 10 mM dextrose, 0.5 mM CaCl_2_, 10 mM MgSO_4_, and 212.7 mM sucrose and transferred to oxygenated (95% O_2_ and 5% CO_2_) artificial cerebrospinal fluid (ACSF) containing 124 mM NaCl, 5 mM KCl, 1.23 mM NaH_2_PO_4_, 2 mM CaCl_2_, 1 mM MgSO_4_, 26 mM NaHCO_3_ and 10 mM dextrose at 28–30°C for at least 1 h before recording. Slices were transferred to a recording chamber where they were perfused continuously with oxygenated ACSF (28–30°C) at a flow rate of 2 ml/min. A hippocampal CA1 field excitatory postsynaptic potential (fEPSP) was evoked by Schaffer collateral stimulation (0.2 ms current pulses) using a concentric bipolar electrode. Synaptic responses were recorded with ACSF-filled microelectrodes (1–3 MΩ) and were quantified as the initial slope of fEPSP in CA1 as described previously^62^. Recordings were performed using an AM-1800 microelectrode amplifier (A-M systems, Sequim, WA), a PG 4000A stimulator (Cygnus Technology, Delaware Water Gap, PA), and a SIU-90 isolated current source (Cygnus Technology, Delaware Water Gap, PA). Baseline responses were collected at 0.07 Hz with a stimulation intensity that yielded a 40–60% maximal response. LTP was induced by four episodes of theta burst stimulation (TBS) with 10 s intervals. TBS consisted of ten stimulus trains delivered at 5 Hz; each train consisted of four pulses at 100 Hz. Data from slices with stable recordings (<5% change over the baseline period) were included in the analysis. All data are presented as the mean ± S.E.M. normalized to the preconditioning baseline (at least 20 min of stable responses). The experimenters were blind to mouse genotypes. IGOR software (Wavemetrics, Lake Oswego, OR) was used for digitizing and analyzing the responses.

To measure cerebellar long-term depression (LTD), parasagittal cerebellar brain slices (250 μm thick) were cut at 2–3°C using a vibrating tissue slicer. Brain slices were incubated for 30 min at 35°C and then kept at room temperature before recording. ACSF used for cutting contained the following (mM): 110 choline chloride, 2.5 KCl, 7 MgCl_2_, 0.5 CaCl_2_, 2.4 sodium-pyruvate, 1.3 sodium-ascorbate, 1.2 NaH_2_PO_4_, 25 NaHCO_3_, and 20 d-glucose. ACSF used for incubating contained the following (mM): 124 NaCl, 2.5 KCl, 1.3 MgCl_2_, 2.5 CaCl_2_, 2.5 NaH_2_PO_4_, 26.2 NaHCO_3_, and 20 d-glucose. Brain slices were placed on an Olympus BX-50WI upright light microscope and perfused with standard ACSF. Whole-cell patch-clamp recordings were obtained from lobules 3–6 in Purkinje cells using an EPC-9 amplifier (HEKA Elektronik) at 30–32°C as described previously^63^. A patch electrode (2.5–3.0 MΩ) was filled with the following (in mM): 135 CsMS, 10 CsCl, 10 HEPES, 0.2 EGTA, 4 Na_2_-ATP, and 0.4 Na_3_-GTP for sEPSC experiments; and 130 K-gluconate, 10 NaCl, 10 HEPES, 0.3 EGTA, 2 MgCl_2_, 4 Na_2_-ATP, 0.4 Na_3_-GTP, and 10 Tri-phosphocreatine for the other experiments. The holding potential was −70 mV in voltage-clamp mode. Recordings were excluded if the series resistance varied by >15% and the injection current for the holding potential exceeded 600 pA. Parallel fiber (PF) inputs were activated by an ACSF-filled electrode in the molecular layer and climbing fiber (CF) inputs in the granule cell layer. LTD was induced by 30 repeats of a pairing stimulation for 5 min in the current-clamp mode. Pairing stimulation consisted of 7 PF stimuli at 100 Hz with 1 CF stimulus delayed by 150 ms. Differences in LTP or LTD between WT and TLR-2 KO mice were assessed by two-way ANOVA analysis on the average values of the last 10 min of the recording.

### Histological and biochemical measurements

#### Tissue preparation, Nissl staining, and immunohistochemistry

Mice were anesthetized with an intramuscular injection of Zoletil 50® (10 mg per kg body weight) and perfused transcardially with phosphate buffer (100 mM, pH 7.4) followed by ice-cold 4% paraformaldehyde (wt/vol). The brains were removed and post-fixed overnight in phosphate buffer (50 mM, pH 7.4) containing 4% paraformaldehyde. The brains were then immersed in a 30% sucrose solution (in 50 mM phosphate-buffered saline, PBS, wt/vol) and stored at 4°C until sectioning. Frozen brains were sectioned along the coronal plane (30 μm) using a cryostat (Leica Microsystems AG, Germany) and maintained in a storage solution at 4°C. For the Nissl staining, sections were mounted onto gelatin-coated slides, and they were stained with 0.5% cresyl violet (wt/vol), dehydrated through graded alcohols (70%, 80%, 90%, and 100% × 2; vol/vol), placed in xylene, and covered with a coverslip after the addition of Histomount media. To quantify the volume of each brain region, the area of each brain region (6 slices per mouse) was measured using a computerized image-analysis system (Leica Microsystems AG, Wetzlar, Germany). For the immunohistochemistry, free-floating sections were incubated for 24 h in PBS (4°C) containing primary antibody, 0.3% Triton X-100 (vol/vol), 0.5 mg/ml bovine serum albumin, and 1.5% normal horse serum (vol/vol). Afterwards, the sections were incubated for 2 h with biotinylated secondary antibody (1:200 dilution) and then with avidin–biotin–peroxidase complex (1:100 dilution) for 1 h at room temperature. The sections were then reacted with 0.02% diaminobenzidine (DAB, wt/vol) and 0.01% H_2_O_2_ (vol/vol) for 3 min. After each incubation step, the sections were washed three times with PBS. Finally, the sections were mounted on gelatin-coated slides, dehydrated in increasing alcohol concentrations, and cleared in xylene.

#### Volumetric study using magnetic resonance imaging (MRI)

MRI images were obtained using a 9.4 T/160 mm animal MRI system (Agilent Technologies, Santa Clara, CA). Radiofrequency excitation and signal detection were accomplished with a 72 mm quadrature volume coil and a two channel phased array coil, respectively. The imaging protocol included a T2-weighted Image (T2WI) (TR = 4500 ms; TE = 80 ms; slice thickness = 0.5 mm; and matrix = 256 × 256 (no gap)). Volumetric analyses of MRI images were performed using the VnmrJ 4.0 software provided with the scanner (Agilent Technologies, Santa Clara, CA) according to the adult mouse brain atlas^64^.

#### Terminal deoxynucleotidyl transferase-mediated deoxyuridine triphosphate-biotin nick end labeling staining

The brain sections were processed for terminal deoxynucleotidyl transferase-mediated deoxyuridine triphosphate-biotin nick end labeling (TUNEL) staining using an ApopTag® *in situ* kit (Chemicon International, Billerica, MA). A DAB substrate kit (Vector Laboratories, Burlingame, CA) was used for peroxidase staining, and the sections were then counterstained with methyl green. Control sections were incubated in the absence of terminal deoxynucleotidyl transferase enzyme. Investigators who were blind to the experimental conditions carried out all TUNEL analyses.

#### Western blotting

Haloperidol, clozapine, or LTA was injected intraperitoneally into mice 1 h before the sacrifice to acquire samples. Mice were sacrificed by decapitation, and the brain was immediately removed to isolate cortex, hippocampus, midbrain, or cerebellum tissue. Isolated tissues were homogenized in ice-chilled Tris-HCl buffer (20 mM, pH 7.4). Samples of homogenates (15 μg of protein) were subjected to SDS-PAGE under reducing conditions. Proteins were transferred onto PVDF membranes in transfer buffer and further separated at 100 V for 2 h at 4°C to determine expression levels. The Western blots were incubated for 2 h with blocking solution (5% skim milk, wt/vol) at 4°C, then overnight with primary antibodies. The blots were then washed twice with Tween 20/Tris-buffered saline (TTBS), incubated with a 1:5000 dilution of horseradish peroxidase-conjugated secondary antibody for 1 h at room temperature, washed three times with TTBS, and developed by enhanced chemiluminescence (Amersham Life Science, Arlington Heights, IL). Immunoblots were imaged using a bio-imaging program on a LAS-4000 mini imager (Fujifilm Lifescience USA, Stamford, CT) and analyzed using Multi Gauge version 3.2 (Fujifilm Holdings Corporation, Tokyo, Japan). The levels of pAkt and pGSK-3α/β were determined by calculating the ratios of phosphorylated protein to the corresponding total protein on the same membranes. These mean values were normalized to the ratios obtained for the WT animals.

### Statistical analysis

All data are shown as the mean ± S.E.M. and were analyzed using unpaired two-tailed Student's *t*-test, one-way analysis of variance (ANOVA) with Tukey's post hoc comparison or two-way ANOVA with Bonferroni's post hoc test. All statistical analyses were performed using GraphPad Prism 5.0 (Graphpad, La Jolla, CA). The statistical analyses are presented in Supplementary Table 1.

## Author Contributions

S.J.P., J.Y.L., J.H.R. and T.Y.Y. designed the research. S.J.P. and J.Y.L. performed the experiments and analyzed the data. S.J.K. and S.C. provided the electrophysiological experiments. S.J.P., J.Y.L., T.Y.Y. and J.H.R. wrote the manuscript.

## Supplementary Material

Supplementary Information Supplementary information

Supplementary Information Supplementary Table

## References

[b1] van Os J. & Kapur S. Schizophrenia. Lancet 374, 635–645 (2009). 19700006 10.1016/S0140-6736(09)60995-8

[b2] Ross C. A. *et al.* Neurobiology of schizophrenia. Neuron 52, 139–153 (2006). 17015232 10.1016/j.neuron.2006.09.015

[b3] Keshavan M. S. & Hogarty G. E. Brain maturational processes and delayed onset in schizophrenia. Dev Psychopathol 11, 525–543 (1999). 10532623 10.1017/s0954579499002199

[b4] Brennand K. J. *et al.* Modelling schizophrenia using human induced pluripotent stem cells. Nature 473, 221–225 (2011). 21490598 10.1038/nature09915 PMC3392969

[b5] Altamura A. C., Pozzoli S., Fiorentini A. & Dell'osso, B. Neurodevelopment and inflammatory patterns in schizophrenia in relation to pathophysiology. Prog Neuropsychopharmacol Biol Psychiatry 42, 63–70 (2013). 23021973 10.1016/j.pnpbp.2012.08.015

[b6] Meyer U., Schwarz M. J. & Muller N. Inflammatory processes in schizophrenia: a promising neuroimmunological target for the treatment of negative/cognitive symptoms and beyond. Pharmacol Ther 132, 96–110 (2011). 21704074 10.1016/j.pharmthera.2011.06.003

[b7] Potvin S. *et al.* Inflammatory cytokine alterations in schizophrenia: a systematic quantitative review. Biol Psychiatry 63, 801–808 (2008). 18005941 10.1016/j.biopsych.2007.09.024

[b8] Connor C. M. *et al.* Maternal immune activation alters behavior in adult offspring, with subtle changes in the cortical transcriptome and epigenome. Schizophr Res 140, 175–184 (2012). 22804924 10.1016/j.schres.2012.06.037 PMC3568668

[b9] Gibney S. M. & Drexhage H. A. Evidence for a dysregulated immune system in the etiology of psychiatric disorders. J Neuroimmune Pharmacol 8, 900–920 (2013). 23645137 10.1007/s11481-013-9462-8

[b10] Anderson K. V. Toll signaling pathways in the innate immune response. Curr Opin Immunol 12, 13–19 (2000). 10679407 10.1016/s0952-7915(99)00045-x

[b11] Kawai T., Akira S. The role of pattern-recognition receptors in innate immunity: update on Toll-like receptors. Nat Immunol 11, 373–384 (2010). 20404851 10.1038/ni.1863

[b12] Okun E. *et al.* Toll-like receptor 3 inhibits memory retention and constrains adult hippocampal neurogenesis. Proc Natl Acad Sci U S A 107, 15625–15630 (2010). 20713712 10.1073/pnas.1005807107 PMC2932590

[b13] Okun E., Griffioen K. J. & Mattson M. P. Toll-like receptor signaling in neural plasticity and disease. Trends Neurosci 34, 269–281 (2011). 21419501 10.1016/j.tins.2011.02.005 PMC3095763

[b14] Rolls A. *et al.* Toll-like receptors modulate adult hippocampal neurogenesis. Nat Cell Biol 9, 1081–1088 (2007). 17704767 10.1038/ncb1629

[b15] Okun E. *et al.* Evidence for a Developmental Role for TLR4 in Learning and Memory. PLoS One 7, e47522 (2012). 23071817 10.1371/journal.pone.0047522 PMC3469493

[b16] Fazel S. *et al.* Schizophrenia and violence: systematic review and meta-analysis. PLoS Med 6, e1000120 (2009). 19668362 10.1371/journal.pmed.1000120 PMC2718581

[b17] Freyberg Z., Ferrando S. J. & Javitch J. A. Roles of the Akt/GSK-3 and Wnt signaling pathways in schizophrenia and antipsychotic drug action. Am J Psychiatry 167, 388–396 (2010). 19917593 10.1176/appi.ajp.2009.08121873 PMC3245866

[b18] Emamian E. S. *et al.* Convergent evidence for impaired AKT1-GSK3β signaling in schizophrenia. Nat Genet 36, 131–137 (2004). 14745448 10.1038/ng1296

[b19] Porsolt R. D., Moser P. C. & Castagne V. Behavioral indices in antipsychotic drug discovery. J Pharmacol Exp Ther 333, 632–638 (2010). 20200119 10.1124/jpet.110.166710

[b20] Ellenbroek B. A. & Cools A. R. Animal models for the negative symptoms of schizophrenia. Behav Pharmacol 11, 223–233 (2000). 11103877 10.1097/00008877-200006000-00006

[b21] Powell S. B., Weber M. & Geyer M. A. Genetic models of sensorimotor gating: relevance to neuropsychiatric disorders. Curr Top Behav Neurosci 12, 251–318 (2012). 22367921 10.1007/7854_2011_195 PMC3357439

[b22] Frantseva M. V. *et al.* Evidence for impaired long-term potentiation in schizophrenia and its relationship to motor skill learning. Cereb Cortex 18, 990–996 (2008). 17855721 10.1093/cercor/bhm151

[b23] Boyden E. S. *et al.* Selective engagement of plasticity mechanisms for motor memory storage. Neuron 51, 823–834 (2006). 16982426 10.1016/j.neuron.2006.08.026

[b24] Vita A., De Peri L., Silenzi C. & Dieci M. Brain morphology in first-episode schizophrenia: a meta-analysis of quantitative magnetic resonance imaging studies. Schizophr Res 82, 75–88 (2006). 16377156 10.1016/j.schres.2005.11.004

[b25] Santos-Sierra S. *et al.* Mal connects TLR2 to PI3Kinase activation and phagocyte polarization. EMBO J 28, 2018–2027 (2009). 19574958 10.1038/emboj.2009.158 PMC2718282

[b26] Oranje B. *et al.* Effects of typical and atypical antipsychotics on the prepulse inhibition of the startle reflex in patients with schizophrenia. J Clin Psychopharmacol 22, 359–365 (2002). 12172334 10.1097/00004714-200208000-00005

[b27] Kapfhamer D. *et al.* Protein Phosphatase 2α and glycogen synthase kinase 3 signaling modulate prepulse inhibition of the acoustic startle response by altering cortical M-Type potassium channel activity. J Neurosci 30, 8830–8840 (2010). 20592205 10.1523/JNEUROSCI.1292-10.2010 PMC3842471

[b28] Janeway C. A. Jr. & Medzhitov R. Innate immune recognition. Annu Rev Immunol 20, 197–216 (2002). 11861602 10.1146/annurev.immunol.20.083001.084359

[b29] Akira S., Uematsu S. & Takeuchi O. Pathogen recognition and innate immunity. Cell 124, 783–801 (2006). 16497588 10.1016/j.cell.2006.02.015

[b30] Hanke M. L. & Kielian T. Toll-like receptors in health and disease in the brain: mechanisms and therapeutic potential. Clin Sci (Lond) 121, 367–387 (2011). 21745188 10.1042/CS20110164 PMC4231819

[b31] Lathia J. D. *et al.* Toll-like receptor 3 is a negative regulator of embryonic neural progenitor cell proliferation. J Neurosci 28, 13978–13984 (2008). 19091986 10.1523/JNEUROSCI.2140-08.2008 PMC2637819

[b32] Okun E. *et al.* TLR2 activation inhibits embryonic neural progenitor cell proliferation. J Neurochem 114, 462–474 (2010). 20456021 10.1111/j.1471-4159.2010.06778.x PMC2910143

[b33] Consortium SWGotPG. Biological insights from 108 schizophrenia-associated genetic loci. Nature 511, 421–427 (2014). 25056061 10.1038/nature13595 PMC4112379

[b34] Arnone D. *et al.* Magnetic resonance imaging studies in bipolar disorder and schizophrenia: meta-analysis. Br J Psychiatry 195, 194–201 (2009). 19721106 10.1192/bjp.bp.108.059717

[b35] Muller N. *et al.* Impaired monocyte activation in schizophrenia. Psychiatry Res 198, 341–346 (2012). 22429483 10.1016/j.psychres.2011.12.049

[b36] Lien Y. J. *et al.* The multidimensionality of schizotypy in nonpsychotic relatives of patients with schizophrenia and its applications in ordered subsets linkage analysis of schizophrenia. Am J Med Genet B Neuropsychiatr Genet 153B, 1–9 (2010). 19326390 10.1002/ajmg.b.30948

[b37] Kang W. S. *et al.* Association between genetic polymorphisms of Toll-like receptor 2 (TLR2) and schizophrenia in the Korean population. Gene 526, 182–186 (2013). 23644137 10.1016/j.gene.2013.04.058

[b38] Jones C. A., Watson D. J. & Fone K. C. Animal models of schizophrenia. Br J Pharmacol 164, 1162–1194 (2011). 21449915 10.1111/j.1476-5381.2011.01386.x PMC3229756

[b39] Miyakawa T. *et al.* Conditional calcineurin knockout mice exhibit multiple abnormal behaviors related to schizophrenia. Proc Natl Acad Sci U S A 100, 8987–8992 (2003). 12851457 10.1073/pnas.1432926100 PMC166425

[b40] Powell C. M. & Miyakawa T. Schizophrenia-relevant behavioral testing in rodent models: a uniquely human disorder? Biol Psychiatry 59, 1198–1207 (2006). 16797265 10.1016/j.biopsych.2006.05.008 PMC3928106

[b41] Buckley P. F., Miller B. J., Lehrer D. S. & Castle D. J. Psychiatric comorbidities and schizophrenia. Schizophr Bull 35, 383–402 (2009). 19011234 10.1093/schbul/sbn135 PMC2659306

[b42] O'Tuathaigh C. M., Kirby B. P., Moran P. M. & Waddington J. L. Mutant mouse models: genotype-phenotype relationships to negative symptoms in schizophrenia. Schizophr Bull 36, 271–288 (2010). 19934211 10.1093/schbul/sbp125 PMC2833123

[b43] Braff D. L. Prepulse inhibition of the startle reflex: a window on the brain in schizophrenia. Curr Top Behav Neurosci 4, 349–371 (2010). 21312406 10.1007/7854_2010_61

[b44] Braff D. L., Geyer M. A. & Swerdlow N. R. Human studies of prepulse inhibition of startle: normal subjects, patient groups, and pharmacological studies. Psychopharmacology (Berl) 156, 234–258 (2001). 11549226 10.1007/s002130100810

[b45] Perry W., Minassian A., Feifel D. & Braff D. L. Sensorimotor gating deficits in bipolar disorder patients with acute psychotic mania. Biol Psychiatry 50, 418–424 (2001). 11566158 10.1016/s0006-3223(01)01184-2

[b46] Swerdlow N. R. *et al.* Realistic expectations of prepulse inhibition in translational models for schizophrenia research. Psychopharmacology (Berl) 199, 331–388 (2008). 18568339 10.1007/s00213-008-1072-4 PMC2771731

[b47] Kent J. S. *et al.* Motor deficits in schizophrenia quantified by nonlinear analysis of postural sway. PLoS One 7, e41808 (2012). 22870250 10.1371/journal.pone.0041808 PMC3411581

[b48] O'Halloran C. J., Kinsella G. J. & Storey E. The cerebellum and neuropsychological functioning: a critical review. J Clin Exp Neuropsychol 34, 35–56 (2012). 22047489 10.1080/13803395.2011.614599

[b49] Gulsuner S. *et al.* Spatial and temporal mapping of de novo mutations in schizophrenia to a fetal prefrontal cortical network. Cell 154, 518–529 (2013). 23911319 10.1016/j.cell.2013.06.049 PMC3894107

[b50] Guo N. *et al.* A sensitive period for GABAergic interneurons in the dentate gyrus in modulating sensorimotor gating. J Neurosci 33, 6691–6704 (2013). 23575865 10.1523/JNEUROSCI.0032-12.2013 PMC6619092

[b51] Hattiangady B. & Shetty A. K. Neural stem cell grafting counteracts hippocampal injury-mediated impairments in mood, memory, and neurogenesis. Stem Cells Transl Med 1, 696–708 (2012). 23197876 10.5966/sctm.2012-0050 PMC3612501

[b52] Lim J. E. *et al.* The effects of MyD88 deficiency on exploratory activity, anxiety, motor coordination, and spatial learning in C57BL/6 and APPswe/PS1dE9 mice. Behav Brain Res 227, 36–42 (2012). 22051943 10.1016/j.bbr.2011.10.027 PMC3242934

[b53] Arbibe L. *et al.* Toll-like receptor 2-mediated NF-kappa B activation requires a Rac1-dependent pathway. Nat Immunol 1, 533–540 (2000). 11101877 10.1038/82797

[b54] Kaidanovich-Beilin O. & Woodgett J. R. GSK-3: Functional Insights from Cell Biology and Animal Models. Front Mol Neurosci 4, 40 (2011). 22110425 10.3389/fnmol.2011.00040 PMC3217193

[b55] Svenningsson P. *et al.* Diverse psychotomimetics act through a common signaling pathway. Science 302, 1412–1415 (2003). 14631045 10.1126/science.1089681

[b56] Takeuchi O. *et al.* Differential roles of TLR2 and TLR4 in recognition of gram-negative and gram-positive bacterial cell wall components. Immunity 11, 443–451 (1999). 10549626 10.1016/s1074-7613(00)80119-3

[b57] Mosienko V. *et al.* Exaggerated aggression and decreased anxiety in mice deficient in brain serotonin. Transl Psychiatry 2, e122 (2012). 22832966 10.1038/tp.2012.44 PMC3365263

[b58] Naert A., Callaerts-Vegh Z. & D'Hooge R. Nocturnal hyperactivity, increased social novelty preference and delayed extinction of fear responses in post-weaning socially isolated mice. Brain Res Bull 85, 354–362 (2011). 21501666 10.1016/j.brainresbull.2011.03.027

[b59] Saxe M. D. *et al.* Ablation of hippocampal neurogenesis impairs contextual fear conditioning and synaptic plasticity in the dentate gyrus. Proc Natl Acad Sci U S A 103, 17501–17506 (2006). 17088541 10.1073/pnas.0607207103 PMC1859958

[b60] Liu X. *et al.* The ameliorating effects of 5,7-dihydroxy-6-methoxy-2(4-phenoxyphenyl)-4H-chromene-4-one, an oroxylin A derivative, against memory impairment and sensorimotor gating deficit in mice. Arch Pharm Res 36, 854–863 (2013). 23543630 10.1007/s12272-013-0106-6

[b61] Lee H. E. *et al.* Ethanolic extract of the seed of zizyphus jujuba var. spinosa ameliorates cognitive impairment induced by cholinergic blockade in mice. Biomol *&* Ther 21, 299–306 (2013). 10.4062/biomolther.2013.043 PMC3819903 24244815

[b62] Seo J. *et al.* Regulation of hippocampal long-term potentiation and long-term depression by diacylglycerol kinase zeta. Hippocampus 22, 1018–1026 (2012). 21069783 10.1002/hipo.20889

[b63] Chae H. G. *et al.* Transient receptor potential canonical channels regulate the induction of cerebellar long-term depression. J Neurosci 32, 12909–12914 (2012). 22973014 10.1523/JNEUROSCI.0073-12.2012 PMC6703793

[b64] Ullmann J. F. *et al.* An MRI atlas of the mouse basal ganglia. Brain Struct Funct 219, 1343–1353 (2014). 23689500 10.1007/s00429-013-0572-0

