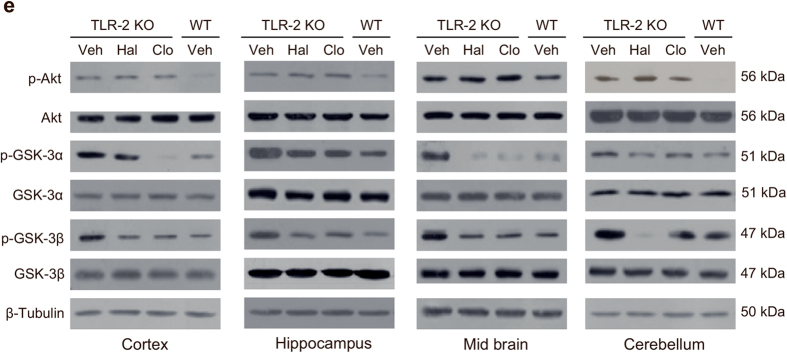# Corrigendum: Toll-like receptor-2 deficiency induces schizophrenia-like behaviors in mice

**DOI:** 10.1038/srep14025

**Published:** 2015-09-18

**Authors:** Se Jin Park, Jee Youn Lee, Sang Jeong Kim, Se-Young Choi, Tae Young Yune, Jong Hoon Ryu

Scientific Reports 5: Article number: 850210.1038/srep08502; published online: 02172015; updated: 09182015

This Article contains an error in Fig. 5e, where the p-Akt immunoblots depicted for the Cerebellum are a duplication of the p-Akt immunoblots depicted for the Hippocampus. The correct Fig. 5e appears below as Fig. 1.

## Figures and Tables

**Figure 1:**