# Supplementary material for: Toll-like receptor-2 deficiency induces schizophrenia-like behaviors in mice
Source: Sci Rep. 2015 Feb 17;5:8502. doi: 10.1038/srep08502 (PMC4330527; doi:10.1038/srep08502)
Supplement: Supplementary Information [file srep08502-s1.doc]

***Supplementary information***

**Toll-like receptor-2 deficiency induces schizophrenia-like behaviors in mice**

Se Jin Park, Jee Youn Lee, Sang Jeong Kim, Se-Young Choi, Tae Young Yune, and Jong Hoon Ryu

**
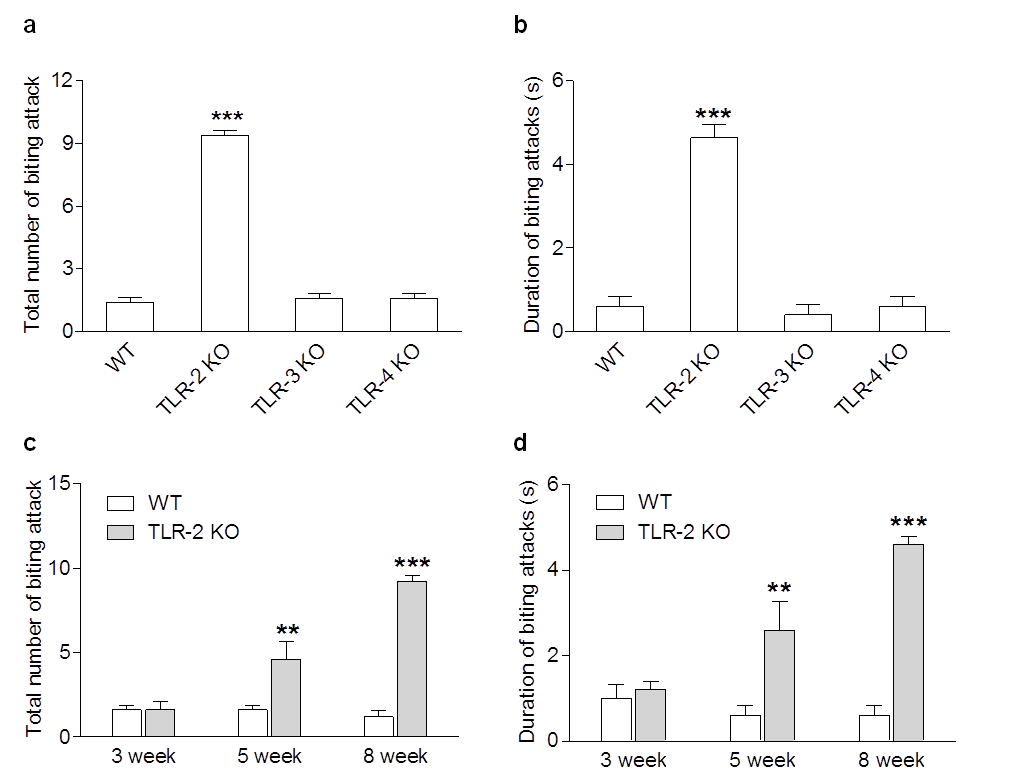
**

**Supplementary Figure 1. Cotton-swab biting test in toll-like receptors (TLRs) knock-out (KO) mice.**

Among TLRs KO mice, only TLR-2 KO mice, but not TLR-3 or TLR-4 KO mice, showed a significant increase in the number of biting attacks (**a**) and the duration of attacks (**b**) against a cotton-swab compared to wild-type (WT) mice. Total number of biting attacks (**c**) and the duration of biting attacks (**d**) were age-dependently increased in TLR-2 KO mice. All data are shown the means ± s.e.m from 5 mice per group. ^**^*P* < 0.01; ^***^*P* < 0.001 vs. WT.


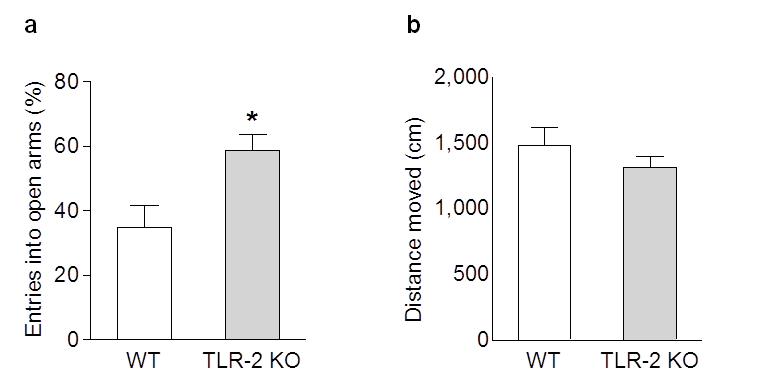


**Supplementary Figure 2. Elevated-plus maze test** **in toll-like receptor (TLR)-2 knock-out (KO) mice.**

(**a**) TLR-2 KO mice (*n* = 7) showed an increased number of entries into the open arms of the elevated-plus maze test compared to wild-type (WT, *n* = 6) mice. (**b**) However, there was no significant difference in the distance moved between WT and TLR-2 KO mice. All data are shown the means ± s.e.m. ^*^*P* < 0.05 vs. WT mice.

**
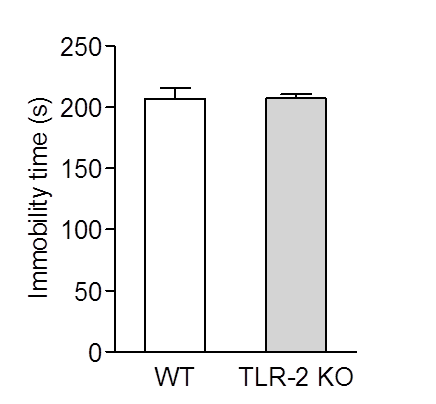
**

**Supplementary Figure 3. Forced swimming test in toll-like receptor (TLR)-2 knock-out (KO) mice.**

Immobility time as an index of depression-like behavior was measured during 6 min in the forced-swimming test. There was no significant difference in immobility times between wild-type (WT, *n* = 8) and TLR-2 knock-out (KO, *n* = 7) mice. Data are shown the means ± s.e.m.

**
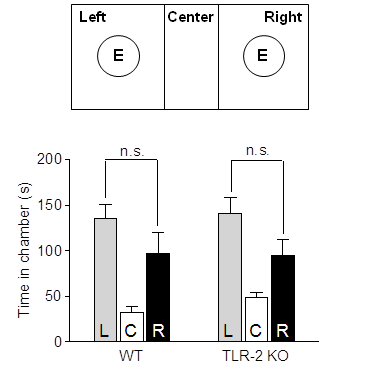
**

**Supplementary Figure 4. Habituation period of the three-chamber social novelty preference test in toll-like receptor (TLR)-2 knock-out (KO) mice.**

During the 5 min habituation trial, empty wire cages (E) were present in the left (L) and right (R) chambers, visible from the middle chamber. The test mouse was placed in the central chamber (C) and explored all three chambers. Both wild-type (WT, *n* = 7) and TLR-2 KO (*n* = 8) mice had no preference for the two chambers during a habituation period. n.s., not significant.


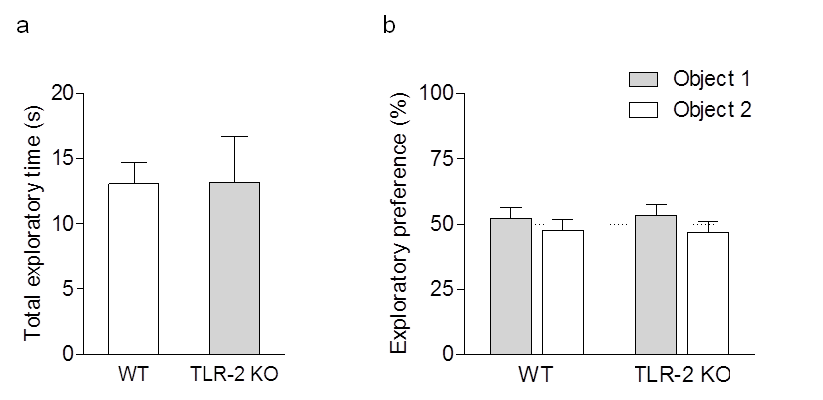


**Supplementary Fig. 5. Exploratory activity during training session of novel object recognition test.**

During training trials, mice were placed in the experimental apparatus in the presence of two identical objects and allowed to explore for 5 min. The duration of time mice spent exploring each object were recorded. WT and TLR-2 KO mice showed similar exploratory time (a) and preference for each object (b). All data are presented as the mean ± s.e.m. (n = 9 per each group).


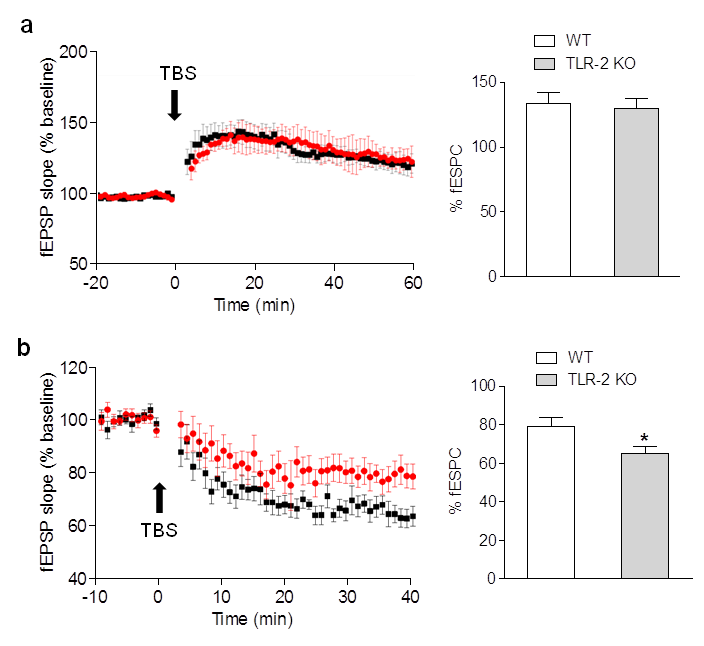


**Supplementary Figure 6.** **Long-term potentiation (LTP) in the hippocampus and long-term depression (LTD) in the cerebellum**.

(**a**) Hippocampal slices prepared from 8-10 week old wild-type (WT) and TLR-2 knock-out (KO) mice were stimulated by theta burst stimulation (TBS) to induce LTP, measured as increases in the slopes of field fEPSPs. Examples of representative field potential traces from WT and TLR-2 KO slices. There were no differences in hippocampal LTP between WT (*n* = 7) and TLR-2 KO mice (*n* = 9). (**b**) LTD was induced by 30 pairing stimulations for 5 min. Cerebellar LTD was inhibited in TLR-2 KO mice (*n* = 12) compared to WT mice (*n* = 9). ^*^*P* < 0.05 vs. WT mice. All data are shown the means ± s.e.m. Black square, WT mice; Red circle, TLR-2 KO mice.

**
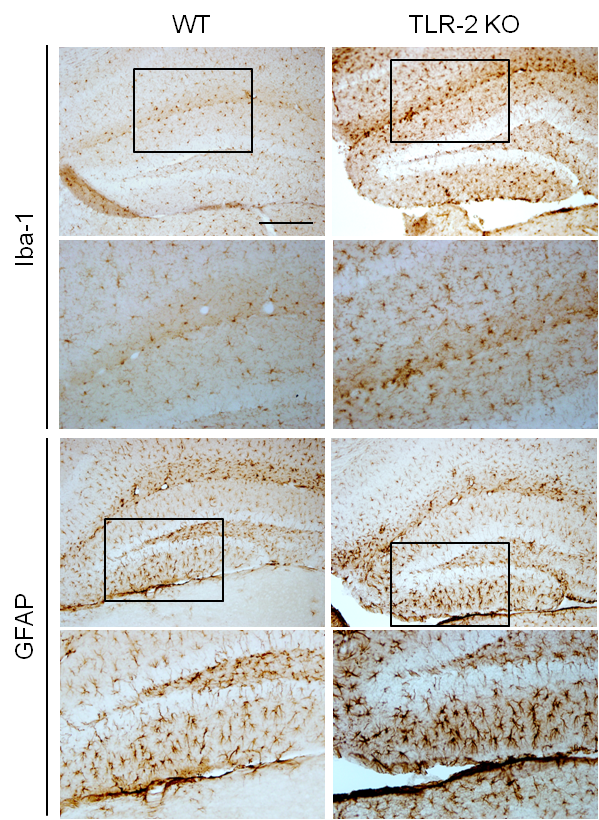
**

**Supplementary Figure 7. Increased intensity of glial cells in the hippocampus of TLR-2 knock-out (KO) mice.**

Representative photograph of wild-type (WT) and TLR-2 KO mouse brains stained with glial cells, including microglia or astrocyte. The numbers of Iba-1-positive or GFAP-positive glial cells in the hippocampal region were higher in TLR-2 KO mice than WT mice. Scale bar, 200 µm. The rectangular subsets were magnified in the rectangle region in each below photomicrograph, respectively.


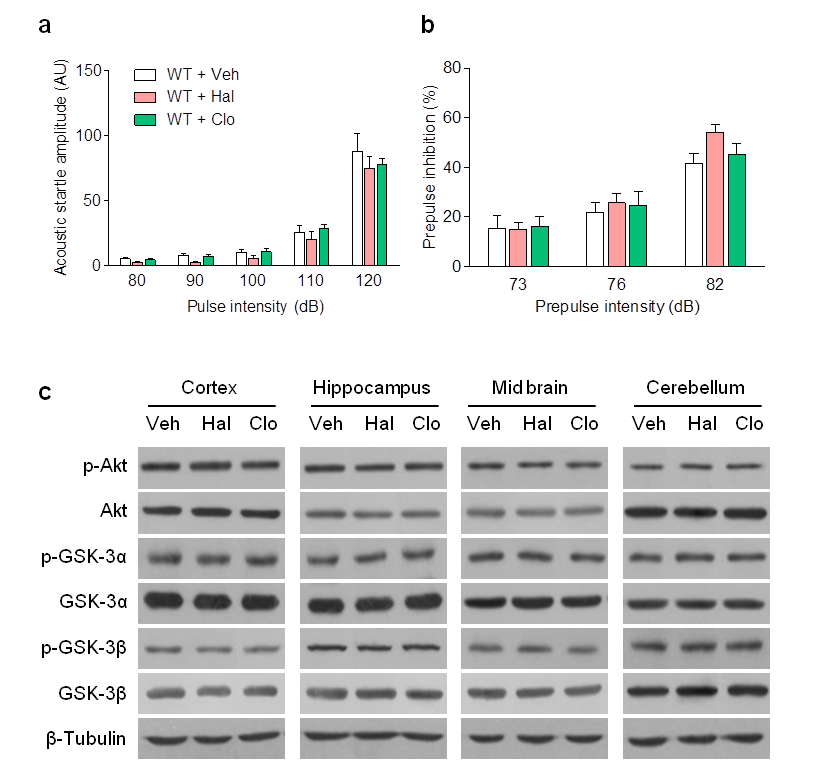


**Supplementary Fig. 8. The effect of haloperidol and clozapine in the wild-type (WT) mice.**

(a, b) In the acoustic startle response test, acute treatment with haloperidol (Hal, 1 mg/kg) or clozapine (Clo, 1 mg/kg) did not affect the level of acoustic startle amplitude and PPI in WT mice. All data are presented as the mean ± s.e.m (*n* = 9 ~ 10 per each group). Veh, vehicle. (c) Immunoblots of p-Akt, p-GSK-3α, and p-GSK-3β were shown in the selected brain regions of WT mouse brains 1 h after the administration of haloperidol or clozapine. The expression levels of p-Akt, p-GSK-3α, and p-GSK-3β in the cortex, hippocampus, midbrain, or cerebellum region were not changed by the acute administration of haloperidol or clozapine compared to the vehicle-treated controls. The gels have been run under the same experimental conditions (n = 3 per each group).


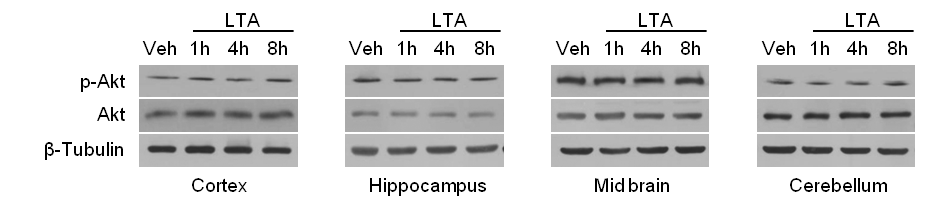


**Supplementary Figure 9. Effects of lipoteichoic acid (LTA) on the expression level of p-Akt and Akt in TLR-2 knock-out (KO) mice.**

To investigate whether lipoteichoic acid (LTA), a TLR-2 ligand, changes the level of p-Akt in TLR-2 KO mice, each brain region including cerebral cortex, hippocampus, midbrain, and cerebellum of TLR-2 KO mice was isolated at the indicated time points (1 h, 4 h, or 8 h, respectively) after the administration of LTA (50 μg, i.p.). Immunoblots were represented the selected brain regions, including cerebral cortex, hippocampus, midbrain, and cerebellum, of TLR-2 KO mouse brains after the administration of LTA. The expression levels of p-Akt or Akt were not changed by the acute administration of LTA in the cortex, hippocampus, midbrain, or cerebellum region compared to the vehicle-treated controls. The gels have been run under the same experimental conditions. Veh, vehicle.
